# *Psy-E1* derived from *Thinopyrum ponticum* contributes strong yellowness to durum wheat but may cause yield loss in Japan

**DOI:** 10.1270/jsbbs.24070

**Published:** 2025-03-26

**Authors:** Keita Kato, Yusuke Ban, Mikiko Yanaka, Motohiro Yoshioka, Hideki Okusu, Tomoki Tanaka, Hiroyuki Kawakami, Masahiro Yamaguchi, Wakako Funatsuki, Kanenori Takata, Miwako Ito

**Affiliations:** 1 Western Region Agricultural Research Center, National Agriculture and Food Research Organization (NARO), Fukuyama, Hiroshima 721-8514, Japan; 2 Kyushu-Okinawa Agricultural Research Center, NARO, Chikugo, Fukuoka 833-0041, Japan; 3 Central Laboratory, NIPPN Corporation, Atsugi, Kanagawa 243-0041, Japan; 4 Tohoku Agricultural Research Center, NARO, Morioka, Iwate 020-0198, Japan; 5 Department of Life and Food Sciences, Obihiro University of Agriculture and Veterinary Medicine, Obihiro, Hokkaido 080-8555, Japan

**Keywords:** durum wheat, yellow pigment, *Psy-A1*·*Psy-E1*·*Lr19*

## Abstract

Strong yellow color, caused by carotenoid accumulation, in semolina flour made from durum wheat (*Triticum turgidum* L. subsp. *durum* (Desf.)) is one of the most important traits for pasta production. The first step in the carotenoid biosynthesis pathway, which is catalyzed by phytoene synthase (PSY), is a bottleneck, and allelic variation of *Psy-A1* in durum wheat produces different yellow pigment contents (YPC) in seeds. Durum wheat carrying leaf rust resistance gene *Lr19*, which was translocated from wheat relative *Thinopyrum ponticum* chromosome 7E to durum wheat chromosome 7A, is known to produce high YPC, and the causal gene is presumed to be *Psy-E1*, which is tightly linked to *Lr19*. In this study, *Psy-E1* produced higher YPC than *Psy-A1* alleles, such as *Psy-A1k*, *Psy-A1l* and *Psy-A1o*, in durum wheat. Segregation analysis demonstrated that *Psy-E1* is located at the *Psy-A1* locus on chromosome 7A. In a 2-year field test of near-isogenic materials, *Psy-E1* was accompanied by yield loss with decreases in grain number per spike, test weight and thousand-kernel weight under moisture conditions typical of wheat-growing areas of Japan. Thus, *Psy-E1* has the potential to contribute high YPC in durum wheat breeding programs, although the applicable cultivation environments are limited.

## Introduction

For pasta products made from semolina flour of durum wheat (*Triticum turgidum* L. subsp. *durum* (Desf.)), yellowness and elastic texture with high protein contents and gluten strength are desirable traits. In particular, a bright yellow color, which is caused by carotenoid yellow pigment content (YPC), is preferred for pasta products (Hentschel *et al.* 2002, Lachman *et al.* 2017). In addition to their role of a visual quality of pasta products, carotenoids have important roles in human nutrition and health owing to their provitamin A activity and antioxidant properties (Abdel-Aal *et al.* 2007, Ficco *et al.* 2014).

YPC of wheat is a stable phenotype influenced by genetics and showing high heritability (Clarke *et al.* 2006, Digesù *et al.* 2009). Recently, many genetic studies have examined carotenoid biosynthesis in plants. This pigment is a mixture of carotenoids, mainly lutein (Digesù *et al.* 2009, Prins *et al.* 1997). Lutein is biosynthesized from geranylgeranyl diphosphate. In *Triticum* species, including durum wheat, lutein accumulates as a yellow pigment in the endosperm. It is converted to phytoene by phytoene synthase (PSY) as the initial step, followed by the downstream reactions of the terpenoid biosynthesis pathway (Ramachandran *et al.* 2010). Phytoene is converted to lycopene in a desaturation step catalyzed by phytoene desaturase (Pds) (Bartley *et al.* 1999) and zeta-carotene desaturase (Zds) (Albrecht *et al.* 1995) and an isomerization step catalyzed by carotenoid isomerase (CRTiso) (Matthews *et al.* 2003). Lycopene is then converted to lutein through cyclization by lycopene-β-cyclase (βLCY) and lycopene-ε-cyclase (εLCY) and hydroxylation by β-hydroxylase (βOHase) and ε-hydroxylase (εOHase) (Fraser and Bramley 2004). In this pathway, three bottlenecks are known in several plants: phytoene synthesis by PSY, lycopene cyclization and carotene hydroxylation (Lindgren *et al.* 2003, Zhou *et al.* 2022). Among these bottlenecks, phytoene synthesis by PSY have been reported as rate-limiting step in wheat (Cong *et al.* 2009).

There are three PSY paralogs in wheat: PSY1 in leaf, which is involved in carotenoid accumulation; PSY2 in green tissues, which is involved in photosynthesis; and PSY3 in root, which regulates ABA biosynthesis under abiotic stress (Dibari *et al.* 2012, Li *et al.* 2008, Pozniak *et al.* 2007, Zhang and Dubcovsky 2008, Zhou *et al.* 2022). Zhang and Dubcovsky (2008) also reported two QTLs related to yellow pigment accumulation, which named *Psy-A1* and *Psy-B1* on chromosome group 7 in durum wheat. Allelic variations in *PSY1* genes were later found in durum wheat and compared their effect for YPC, the effect order the YPC from high to low was *Psy-A1o*, *Psy-A1l* and *Psy-A1a* by recombination inbred lines (Singh *et al.* 2009). In other studies, *Psy-A1o* and *Psy-A1l* had the same effect on YPC and both are stronger than *Psy-A1a* (Campos *et al.* 2016).

Wild relatives of wheat, especially *Thinopyrum ponticum* (syn: *Th. elongatum*, *Agropyron elongatum* and *Lophopyrum* *elongatum*), have been shown to be a resource for leaf rust resistance in breeding programs (McCallum *et al.* 2016, Prasad *et al.* 2020). One leaf rust resistance gene, *Lr19*, has been transferred from *A. elongatum* (*Th. ponticum*) on chromosome 7E to the long arm of wheat chromosome 7D. A translocated segment containing *Lr19* was associated with higher biomass and grain yield under irrigated conditions and with yellow endosperm designated causal gene as *Y* (Monneveux *et al.* 2003, Singh *et al.* 1998). Zhang *et al.* (2005) and Gennaro *et al.* (2009) generated durum wheat–*Th. ponticum* recombinant lines in which a segment containing *Lr19* with *Y* was translocated to chromosome 7A in durum wheat.

*Psy-E1*, the orthologue of *Psy-1* was presumed as candidate gene of *Y* locus derived from *Th. ponticum*, and it was involved in YPC (Zhang and Dubcovsky 2008). The genetic distance between *Lr19* and *Psy-E1* was estimated to be 9.8 cM (Xu *et al.* 2023). Also, unidentified *Psy* paralog adjacent to *Psy-E1* was speculated by the apparent widespread conserved duplication on chromosome group 7 in the *Poaceae* (Ceoloni *et al.* 2014, Gallagher *et al.* 2004, Singh *et al.* 2009).

It has been reported that *Lr19*-linked *Psy-E1* confers high YPC in durum wheat (Xu *et al.* 2023). However, effectiveness of *Psy-E1* on durum wheat breeding program has not been reported so far. In this study, we evaluated *Psy-E1*’s contribution for high YPC and its effect on agronomic traits under Japanese climate condition comparison with the original *Psy-A1* alleles in durum wheat, and possibility of *Psy-E1* and *Psy-A1* pyramiding to increase YPC.

## Materials and Methods

### DNA sequencing of *Psy-A1* gene in durum cultivar ‘Setodure’

DNA was extracted by the potassium acetate method (Dellaporta *et al.* 1983). DNA templates were amplified in a PCR Thermal Cycler Dice (TaKaRa Bio, Shiga, Japan) with LA Taq and 10× LA PCR Buffer II (TaKaRa Bio) and primers listed in Supplemental Table 1. PCR amplification of the open reading frame of *Psy-A1* was an initial 94°C for 1 min, followed by 35 cycles of 95°C for 10 s and 72°C for 10 min. The amplified PCR fragments were separated by agarose gel electrophoresis, visualized with Gel Red stain (Biotium, Fremont, CA, USA) and extracted with a Qiaquick Gel Extraction Kit (Qiagen, Hilden, Germany). Cycle sequencing reactions were performed in a PCR Thermal Cycler Dice with BigDye Terminator v. 3.1 with sequencing primers listed in Supplemental Table 1 and purified with BigDye Terminator (Applied Biosystems, Waltham, MA, USA) following the manufacturer’s instructions. DNA was sequenced on a 3130xl Genetic Analyzer (Applied Biosystems).

### Plant materials and cultivation conditions

To develop near-isogenic lines (NILs), we used the durum wheat cultivar ‘Setodure’ (possessing *Psy-A1k*, see Results) as the recurrent parent (Yanaka *et al.* 2018). Durum wheat cultivars ‘AC Navigator’ (Clarke *et al.* 2000) and ‘Enterprise’ (Singh *et al.* 2010), and experimental line Ap1-22 (Zhang *et al.* 2005), which possess *Psy-A1l*, *Psy-A1o* and *Lr19*, respectively, were used as donors. Experimental line Ap1-22 had translocated chromosome 7E derived from *Th. ponticum* including *Psy-E1* and *Lr19* which approximately 31.7 Mb apart and existed estimated 511 genes (Wang *et al.* 2020). By backcrossing each of the donors to ‘Setodure’, we developed BC_6_F_2_ plants containing each gene of interest by DNA marker-assisted selection. And NIL possessing *Lr19* was designated name as *Psy-E1* NIL.

The three NILs and ‘Setodure’ were grown in a research field at the Western Region Agricultural Research Center (WARC), Fukuyama, Hiroshima, Japan (lat. 34°30′4″N, lng. 133°23′12″E). Their 756 seeds were sown in a strip 0.7 m wide × 7.2 m long on November 11^th^ and harvested in June. There were 4 replicates of materials in two seasons, 2020–21 and 2021–22 (later referred to as harvest years 2021 and 2022, respectively).

### *Psy-A1*, *Psy-E1* and *Lr19* genotyping

DNA was extracted as described above. All PCR reactions used Thermal Cycler Dice with Quick Taq HS (Toyobo, Osaka, Japan), and the amplified PCR fragments were separated and visualized as above. PCR amplification of *Psy-A1* alleles (*Psy-A1k*, *Psy-A1l* and *Psy-A1o*) was an initial 95°C for 2 min, followed by 35 cycles of 94°C for 30 s, 65°C for 30 s, and 68°C for 2 min. Amplification of *Psy-E1* was an initial 95°C for 2 min; 10 cycles with a touchdown step of 94°C for 30 s, 65–56°C for 30 s with a decrease of 1°C per cycle, and 68°C for 1.5 min; and then 25 cycles of 94°C for 30 s, 55°C for 30 s and 68°C for 1.5 min. Amplification of *Lr19* was an initial 95°C for 2 min; 8 cycles with a touchdown step of 94°C for 30 s, 68–61°C for 30 s with a decrease of 1°C per cycle, and 68°C for 1 min; and then 22 cycles of 94°C for 30 s, 60°C for 30 s and 68°C for 1 min. The multiplex PCR of *Psy-A1* and *Psy-E1* with HotStarTaq *Plus* (Qiagen) was an initial 95°C for 5 min, followed by 35 cycles of 94°C for 1 min, 65°C for 1 min, and 72°C for 1 min. *Lr19* with *Waxy-B1* as an internal control was amplified as for genotyping of *Lr19* alone. Primers were listed in Supplemental Table 1, and DNA and deduced amino acid sequences among 3 *Psy-A1* alleles and *Psy-E1* were aligned and showed in Supplemental Figs. 1 and 2.

### Segregation analysis

Segregation ratios of *Psy-A1* and *Psy-E1* were calculated in three F_2_ populations: *Psy-E1* NIL × ‘Setodure’ (*Psy-A1k*), *Psy-E1* NIL × *Psy-A1l* NIL and *Psy-E1* NIL × *Psy-A1o* NIL. Genotyping was performed by multiplex PCR of *Psy-A1* and *Psy-E1*.

### Whole grain, straight flour and semolina flour production

To produce whole-grain, 30 g of dried seeds were pulverized in a cyclone mill (Cyclotec 1093; Foss, Hillerød, Denmark) with a 0.5-mm sieve.

To produce straight flour by small-scale milling, 150 g of dried seeds were milled using a Quadrumat Jr. (C.W. Brabender Instruments, Inc., South Hackensack, NJ, USA) without a sieve. The milled samples were sieved in a rotary sifter with a 355-μm sieve for 5 min at 360 rpm, and the material that passed through the sieve was used as straight flour.

To produce semolina flour by large-scale milling, seed from 4 replicates was combined, and 2 kg of seeds were milled in a Bühler Laboratory Mill (Bühler, Uzwil, Switzerland) following Cereal & Grains Association method AACC 26-41.01 (AACC Approved Methods of Analysis 1999b).

### Pasta production

Pasta processing using semolina flour in 2021 and the boiling test were performed following AACC 66-42.01 (AACC Approved Methods of Analysis 1999c) and AACC 66-50.01 (AACC Approved Methods of Analysis 1999d), respectively.

### Yellow pigment content (YPC) quantification and b* value of CIELAB measurement

YPC was extracted from whole grain, straight flour, and semolina flour by AACC 14-50.01 (AACC Approved Methods of Analysis 1999a) and quantified on a Nanodrop 2000c spectrophotometer (Thermo Fisher Scientific, Waltham, MA, USA). The b* value of the CIELAB color space, indicative of yellow color intensity, was measured on a CM3500d spectrophotometer (Konica Minolta, Inc., Tokyo, Japan) with light source C and a 2° field for whole grain, straight flour, and semolina flour, and spectrophotometer CM-5 (Konica Minolta) with light source C and a 2° field for dried and boiled spaghetti. The dried spaghetti pieces were lined up without gaps and measured. Spaghetti that had been boiled for 5 min was packed in a transparent plastic bag and measured the next day.

### Measurement of agronomic traits

Agronomic traits were assessed in 2020 and 2021 following the International Union for the Protection of New Varieties of Plants (UPOV) guidelines (https://www.upov.int/edocs/tgdocs/en/tg003.pdf). Heading date and maturity date were determined as the accumulated days after sowing. Culm length from the base of the plant to the panicle base was measured by ruler and averaged from 10 culms in each of the 4 replicates. Yield was measured from yielding of 0.7 m wide × 7.2 m long and calculated as kg/a. Spike number per square meter was counted manually and averaged from two areas within each of the 4 replicates. Spikes and grains were harvested from each plot. Awn and spike length were measured with a ruler, and spikelet and grains number per spike were counted manually. Spikelet density was calculated based on spikelet number and spike length. These spike traits were averaged from 10 spikes in each of the 4 replicates. Grain protein content and test weight were analyzed with a near-infrared spectrophotometer (IM9500; Perten Instruments, Stockholm, Sweden). Grain diameter, thousand-kernel weight and grain hardness were measured with a single-kernel characterization system (SKCS4100; Perten Instruments).

### Statistical analysis

The YPC and b* values of ‘Setodure’ and NILs were analyzed by ANOVA and Tukey’s HSD at *P* < 0.05. For segregation analysis of *Psy-E1* and *Psy-A1*, chi-squared analysis was applied with two models. By the same-locus model, the expected segregation ratio for homozygous *Psy-E1* : heterozygous *Psy-E1*/*Psy-A1* : homozygous *Psy-A1* was 1:2:1. By the different-locus model, the expected segregation ratio for was both of them : only *Psy-E1* : only *Psy-A1* : null of them as 9:3:3:1. All statistics were analyzed in R version 4.2.0 software (R Core Team 2023).

## Results

### Marker development for genotype determination and segregation analysis

When the genotype of *Psy-A1* in ‘Setodure’ was examined by DNA sequencing with primer walking, it was a complete match to *Psy-A1k* (FJ393522) in *T. turgidum* subsp. *dicoccoides* and to FJ393527 in *T. aestivum* subsp. *Spelta*. Thus, the sequence in ‘Setodure’ is classified as *Psy-A1k* (registered as LC778135).

**Fig. 1.**
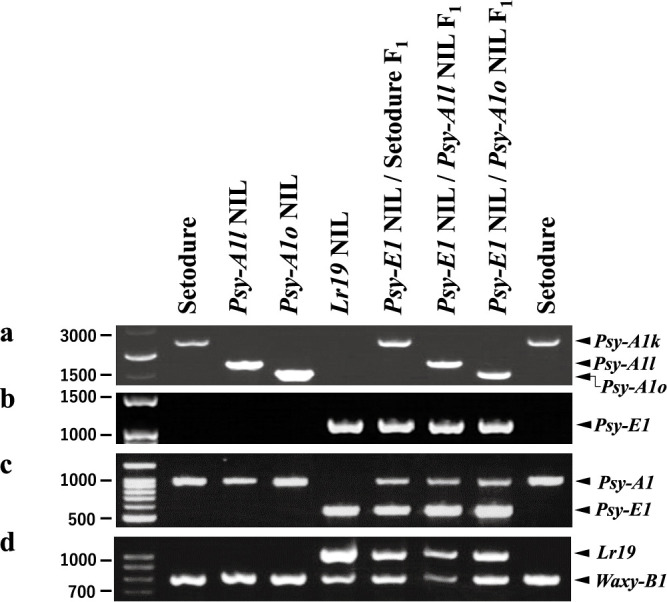
Genotyping of materials containing *Psy-A1*, *Psy-E1* and *Lr19*. Templates were ‘Setodure’, 3 NILs, and 3 F_1_ hybrids of *Psy-E1* NIL × ‘Setodure’ and of *Psy-E1* NIL × *Psy-A1* NILs. a Codominant marker detecting *Psy-A1* alleles. b Dominant *Psy-E1*–specific marker. c Codominant marker detecting *Psy-A1* and *Psy-E1*. d Dominant *Lr19*–specific marker with *Waxy-B1* as internal control.

The *Psy1* genotypes of ‘Setodure’ (*Psy-A1k*), *Psy-A1l* NIL and *Psy-A1o* NIL were clearly distinguished by a codominant DNA marker for *Psy-A1* alleles, but not *Psy-E1* (Fig. 1a). *Psy-E1* NIL was clearly distinguished by a dominant DNA marker for *Lr19* (Fig. 1d) and amplified with a novel *Psy-E1* gene-specific DNA marker (referred to as EU096095 in *Th. ponticum*), but not *Psy-A1*, under both simplex and multiplex PCR conditions (Fig. 1b, 1c). F_1_ progenies crossing *Psy-E1* NIL with ‘Setodure’, *Psy-A1l* NIL or *Psy-A1o* NIL were amplified same fragments with each parent (Fig. 1a–1d).

### YPC quantification and b* value of CIELAB measurement

**Fig. 2.**
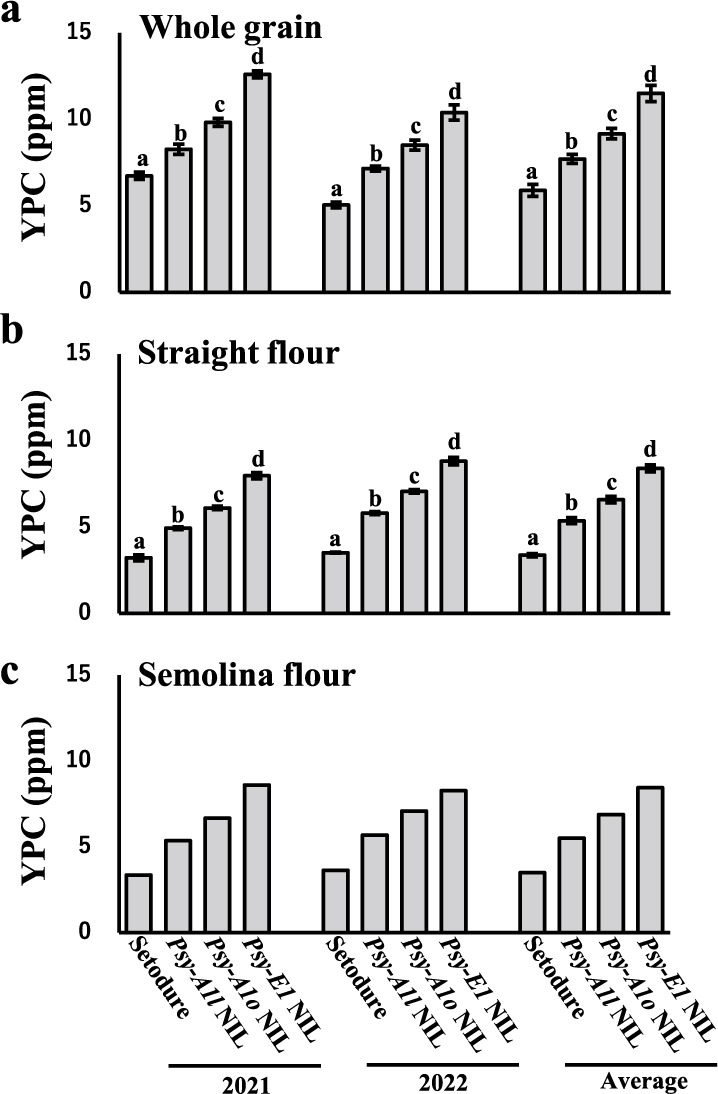
Yellow pigment content (YPC) of ‘Setodure’ and 3 NILs in 2020, 2021 and 2-year average. Sample type was (a) whole grain flour, (b) straight flour and (c) semolina flour. Values were means ± SE (*n* = 4 in 2021 and 2022, *n* = 8 in 2-year average). Same letters in a and b indicate no significant differences by Tukey’s HSD at *P* < 0.05.

**Fig. 3.**
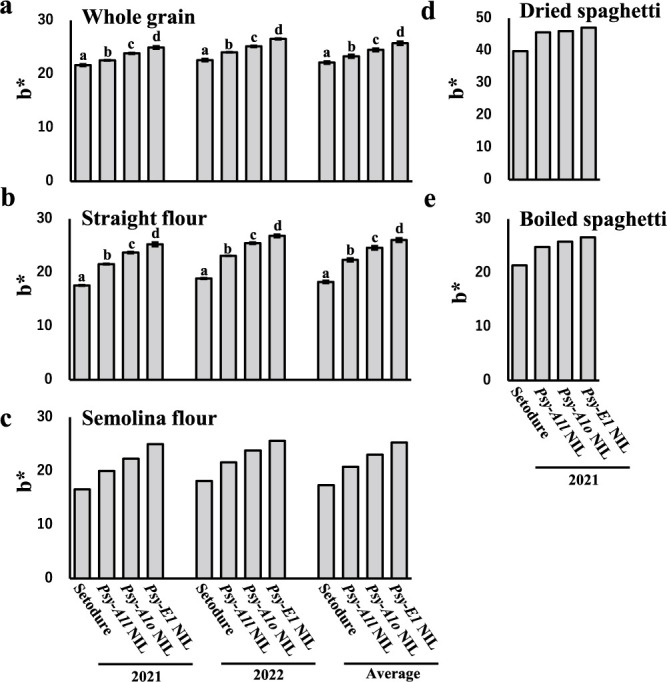
b*value of ‘Setodure’ and 3 NILs in 2020, 2021 and 2-year average. Sample type was (a) whole grain, (b) straight flour, (c) semolina flour, (d) dried spaghetti and (e) boiled spaghetti. Values are means ± SE (a–c: *n* = 4 in 2021 and 2022, *n* = 8 in 2-year average) and mean (d, e: 4 replicates were mixed together in). Same letters in a and b indicate no significant differences by Tukey’s HSD at *P* < 0.05.

To assess the effect of *Psy* genotype on yellowness of flour and pasta, we measured YPC and the b* value of the CIELAB color space in ‘Setodure’ and the three NILs. YPC and b* were the highest in *Lr19* NIL and lowest in ‘Setodure’ (Figs. 2, 3). For both YPC and b*, the genotype effects in order from high to low were *Psy-E1* NIL > *Psy-A1o* NIL > *Psy-A1l* NIL > ‘Setodure’ (*Psy-A1k*), and there were significant differences among the genotypes regardless of sample type (whole grain or straight flour) and for both single years and 2-year averages (Figs. 2a, 2b, 3a, 3b).

YPC and b* in semolina flour in 2021, 2022 and the 2-year average (Figs. 2c, 3c, Supplemental Fig. 3c) and b* in dried and boiled spaghetti in 2021 (Fig. 3d, 3e, Supplemental Fig. 3d) were obtained without replication because large amounts of seed were needed for large-scale milling to obtain semolina flour for making pasta. The orders of the four genotypes were the same for YPC and b* in semolina flour and for b* in dry and boiled spaghetti as those for whole grain and straight flour.

### Segregation analysis

To test our hypothesis that *Psy-E1* lies at the same locus as *Psy-A1*, we analyzed segregation ratios in three F_2_ populations produced by crossing *Psy-E1* NIL with ‘Setodure’ (*Psy-A1k*), *Psy-A1l* NIL or *Psy-A1o* NIL by chi-squared analysis with same- and different-locus models. *Psy-A1*- and/or *Psy-E1*-specific fragments were amplified in all F_2_ individuals (Tables 1, 2), none of any F_2_ individuals amplified neither *Psy-A1*- nor *Psy-E1*-specific fragments (Table 2). In three F_2_ populations produced by crossing *Psy-E1* NIL with ‘Setodure’ (*Psy-A1k*), *Psy-A1l* NIL or *Psy-A1o* NIL, both fragments of *Psy-E1* and *Psy-A1* were amplified only on *Psy-E1*/*A1* heterozygous (Fig. 1c). No significant difference from the expected 1:2:1 ratio was shown for any F_2_ population in the same-locus model (Table 1), whereas significant differences from the expected 9:3:3:1 ratio were shown for all three F_2_ populations in the different-locus model (Table 2). These results show that *Psy-E1* and *Psy-A1* lie at the same locus. *Lr19* could be amplified in individuals heterozygous for *Psy-A1* and *Psy-E1* or homozygous for *Psy-E1*, but not those homozygous for *Psy-A1*. This result is consistent with earlier studies showing that *Lr19* is closely linked to *Psy-E1*, and the linkage was unbroken in this analysis (data not shown) although other studies have shown that these genes can be separated (see Discussion).

### Agronomic trait measurement

Our study was conducted in Japan, where the period of wheat maturation to harvest overlaps with the rainy season with moisture stress.

To evaluate the effects of *Psy* genotype on phenotypes other than yellowness, we tested the characteristics and performance of ‘Setodure’ and the NILs in a 2-year field trial. No significant differences were seen among ‘Setodure’, *Psy-A1l* NIL and *Psy-A1o* NIL in any of the investigated agronomic traits (Table 3). Yield, grain number, test weight and thousand-kernel weight were significantly lower in *Psy-E1* NIL than in the other lines (Table 3), whereas culm length and protein content of *Psy-E1* NIL were significantly higher. Other traits were not significantly different between *Psy-E1* NIL and the other NILs (Supplemental Fig. 3a, 3b).

## Discussion

The chromosome segment containing *Lr19*, derived from the wild relative *Th. ponticum*, confers resistance to leaf rust in wheat (Gennaro *et al.* 2009) and increases the yellowness in seeds, but the degree of its effect on yellowness in durum wheat was unknown. In this study, yellowness of flour and pasta as measured by YPC and b* values in multiple years and in different sample types decreased in the order of *Psy-E1* NIL > *Psy-A1o* NIL > *Psy-A1l* NIL > ‘Setodure’ (*Psy-A1k*), (Figs. 2, 3). Singh *et al.* (2009) reported that b* value decreased in the order of *Psy-A1l* > *Psy-A1o* > *Psy-A1a* in two panels as modern cultivars and landraces in durum wheat, but no significant difference between *Psy-A1l* and *Psy-A1o* under some conditions. *Lr19*-possessing lines were generated in several studies, and their seeds and flour showed strong yellowness (Prins *et al.* 1997, Rai *et al.* 2019, Zhang *et al.* 2005). This is the first report that the yellowness conferred by *Lr19* is higher than that conferred by any of the *Psy-A1* alleles in durum wheat.

*Lr19* is linked with *Y* on chromosome 7E from *Th.* *ponticum*, which confers yellow pigmentation in endosperm (Ayala-Navarrete *et al.* 2013, Sharma and Knott 1966, Zhang and Dubcovsky 2008). Here, *Psy-E1* NIL had high YPC and carries *Lr19* and *Psy-E1* (Figs. 1–3), consistent with previous research. Interestingly, *Psy-E1* NIL did not show amplification of any *Psy-A1*–specific amplicons and only F_1_ crossing *Psy-E1* NIL with ‘Setodure’ (*Psy-A1k*) or *Psy-A1* NILs show both *Psy-A1*–specific and *Psy-E1*–specific amplicons (Fig. 1). In addition, both fragments of *Psy-E1* and *Psy-A1* were detected only on *Psy-E1*/*A1* heterozygous in F_2_ populations. Taking these facts into consideration, we clarified by using segregation analysis that *Psy-E1* is not tandem arrangement with *Psy-A1* but is located at the *Psy-A1* locus (Table 1). These results provide further evidence that *Psy-E1* is a candidate for the *Y* gene linked with *Lr19*.

We observed strong linkage between *Lr19* and *Psy-E1*, consistent with previous research (Knott 1980, Marais and Marais 1990). This linkage block was not broken in this study (Table 1). However, Prins *et al.* (1997) revealed that white-endosperm lines with *Lr19* lacked the *Y* locus from a translocated segment. Xu *et al.* (2023) also succeeded in breaking this linkage with homoeologous recombination and established a white-endosperm line lacking *Psy-E1* but carrying *Lr19*. Wheat breeders would have to choose *Psy-E1* or *Psy-A1* alleles for controlling yellowness, as *Psy-E1* is high likely to locate at same locus as *Psy-A1*.

Here, *Psy-E1* NIL had both higher culm length and protein content, and lower yield than the *Psy-A1*–containing lines, with lower grain number per spike, test weight and thousand-kernel weight than the recurrent durum wheat parent ‘Setodure’ and *Psy-A1* NILs in the ‘Setodure’ genetic background (Table 3). The results of negative correlation of yield and protein content in seeds was consistent with its relationship in cereals (Simmonds 1995). In bread wheat, yield was increased in *Lr19*-containing NILs in various backgrounds in India (Rai *et al.* 2019) and Mexico (Reynolds *et al.* 2001). The yield and number of grains per m^2^ were increased in *Lr19*-containing NILs under appropriate irrigated conditions compared to under drought conditions (Monneveux *et al.* 2003) and yield increase with biomass and number of spikes per m^2^ elevate was observed under non-moisture conditions with appropriate irrigations compared to under moisture conditions (Singh *et al.* 1998). They concluded that the translocation of chromosome 7E may be useful for enhancing yield under favorable conditions (Monneveux *et al.* 2003, Singh *et al.* 1998). Monneveux *et al.* (2003) pointed out that agronomic phenotypes of the effect of the chromosome 7E translocation depend on the recipient genotype. Thus, the agronomic potential and effectiveness of the 7E segment including *Lr19* and *Psy-E1* will need to be assessed under appropriate cultivation conditions and in various durum wheat backgrounds.

Functional characterization of PSY in carotenogenesis has revealed various regulatory stages acting at the transcriptional, post-transcriptional, post-translational and epigenetic levels (Zhou *et al.* 2022). The effects of engineering the carotenoid biosynthetic pathway through manipulation of *Psy1* have been researched in many plants, because PSY is a highly conserved enzyme that catalyzes the first step in the carotenoid biosynthesis pathway and is a major rate-limiting enzyme (Cong *et al.* 2009, Lindgren *et al.* 2003, Zhou *et al.* 2022).

Alternative splicing occurs in *Psy-A1* owing to duplication in an exon, producing aberrant transcripts and decreasing the yellow color of endosperm in bread wheat (Howitt *et al.* 2009). Cultivars of durum and bread wheat with strong yellow endosperm had higher *Psy1* gene expression (Qin *et al.* 2016). Tissue-specific expression and overexpression of bacterial PSY genes dramatically increased provitamin A in bread wheat (Wang *et al.* 2014). However, functional characterization of *Psy-A1* alleles and *Psy-E1* in wheat is still incomplete, and the effects of these alleles need to be better understood to lead to effective durum wheat breeding in the future.

In this study, *Lr19*-linked *Psy-E1* conferred higher yellowness in endosperm, a trait desirable for pasta production. However, it was accompanied by yield loss under the field conditions in this study, which were typical for wheat-growing areas of Japan. Among the future challenges for durum wheat breeding will be to break the linkage between *Psy-E1* and yield loss to enable application of *Psy-E1* in breeding programs, and to explore the favorable combination of *Psy-A1* and *Psy-B1* in durum wheat to produce yellowness as strong as that conferred by *Psy-E1*. Although *Psy-B1* allele was still unknown in ‘Setodure’, the effect of *Psy-B1* alleles may increase YPC. For *Psy-B1* alleles in durum wheat, *Psy-B1a* and *Psy-B1b* had the same effect on YPC (Campos *et al.* 2016), and *Psy-B1f* had a stronger effect on YPC than *Psy-B1g* (He *et al.* 2009).

## Author Contribution Statement

KK: Conceptualization, data curation, investigation, resources, validation, visualization, writing original draft. YB and MI: Data curation, investigation, validation. MY: Data curation, MY, WF and KT: Resources. HO, TT, HK and MY: Data curation, validation. All authors read and approved the final manuscript.

## Supplementary Material

Supplemental Figures

Supplemental Table

## Figures and Tables

**Table 1.** Segregation analysis with same-locus model in F_2_ populations of *Psy-E1* NIL crossed with ‘Setodure’ or *Psy-A1* NILs

Cross	N	Segregation			Chi-squared value*	*P*-value
		*Psy-E1* homozygous	*Psy-E1*/*Psy-A1* heterozygous	*Psy-A1* homozygous		
*Psy-E1* NIL × Setodure (*Psy-A1k*)	267	71	122	74	2.049	0.359
*Psy-E1* NIL × *Psy-A1l* NIL	237	59	114	60	0.116	0.944
*Psy-E1* NIL × *Psy-A1o* NIL	258	56	135	67	1.412	0.493

* Expected ratio of *Psy-E1* homozygous : *Psy-E1*/*Psy-A1* heterozygous : *Psy-E1* homozygous = 1:2:1 was tested by chi-squared test.

**Table 2.** Segregation analysis with different-locus model in F_2_ population of *Psy-E1* NIL crossed with ‘Setodure’ or *Psy-A1* NILs

Cross	N	Segregation				Chi-squared value*	*P*-value
		Both *Psy-E1* and *A1*	Only *Psy-E1*	Only *Psy-A1*	Neither *Psy-E1* nor *A1*		
*Psy-E1* NIL × Setodure (*Psy-A1k*)	267	122	71	74	0	42.180	3.67E-09
*Psy-E1* NIL × *Psy-A1l* NIL	237	114	59	60	0	28.242	3.23E-06
*Psy-E1* NIL × *Psy-A1o* NIL	258	135	56	67	0	25.204	1.40E-05

* Expected ratio of both *Psy-E1* and *Psy-A1* : only *Psy-E1* : only *Psy-A1* : neither *Psy-E1* nor *Psy-A1* = 9:3:3:1 was tested by chi-squared test.

**Table:** Field traits

	Heading date*^a^* (Days)	Maturity date*^a^* (Days)	Spike number (/m^2^)	Yield (kg/10a)	Culm length (cm)
2021					
Setodure	154 ± 0.3 a	203 ± 0.4 a	453 ± 16.0 a	55.1 ± 0.61 a	82 ± 0.6 a
*Psy-A1l* NIL	153 ± 0.3 a	203 ± 0.3 a	454 ± 19.3 a	61.1 ± 2.66 a	80 ± 0.8 a
*Psy-A1o* NIL	153 ± 0.3 a	203 ± 0.3 a	471 ± 37.0 a	54.5 ± 2.35 a	81 ± 0.8 a
*Psy-E1* NIL	154 ± 0.0 a	204 ± 0.3 a	447 ± 23.2 a	48.0 ± 1.00 b	85 ± 1.1 b
2022					
Setodure	147 ± 0.5 a	202 ± 0.3 a	414 ± 11.9 a	51.7 ± 0.99 a	84 ± 0.9 a
*Psy-A1l* NIL	145 ± 0.6 a	201 ± 0.5 a	401 ± 13.3 a	56.2 ± 1.04 a	83 ± 0.8 a
*Psy-A1o* NIL	146 ± 0.3 a	201 ± 0.3 a	433 ± 20.8 a	54.5 ± 2.31 a	85 ± 1.2 a
*Psy-E1* NIL	147 ± 0.5 a	201 ± 0.3 a	415 ± 33.1 a	43.3 ± 1.26 b	91 ± 0.8 b

**Table:** Spike traits

	Spike length (cm)	Awn length (cm)	Spikelet number (/spike)	Spikelet density*^b^*	Grain number (/spike)
2021					
Setodure	9.2 ± 0.22 a	6.9 ± 0.44 a	23 ± 0.5 a	2.5 ± 0.08 a	74 ± 2.4 a
*Psy-A1l* NIL	9.1 ± 0.11 a	6.0 ± 0.25 a	25 ± 0.6 a	2.7 ± 0.08 a	73 ± 2.0 a
*Psy-A1o* NIL	9.0 ± 0.31 a	7.5 ± 0.42 a	22 ± 0.6 a	2.4 ± 0.07 a	74 ± 2.3 a
*Psy-E1* NIL	9.3 ± 0.35 a	6.6 ± 0.39 a	24 ± 0.7 a	2.6 ± 0.13 a	63 ± 1.3 b
2022					
Setodure	9.2 ± 0.36 a	6.9 ± 0.54 a	23 ± 0.4 a	2.6 ± 0.11 a	71 ± 3.1 a
*Psy-A1l* NIL	9.5 ± 0.23 a	6.4 ± 0.56 a	24 ± 0.5 a	2.6 ± 0.06 a	73 ± 1.8 a
*Psy-A1o* NIL	9.2 ± 0.32 a	7.3 ± 0.35 a	22 ± 0.3 a	2.4 ± 0.12 a	73 ± 2.0 a
*Psy-E1* NIL	9.0 ± 0.22 a	6.2 ± 0.48 a	24 ± 0.5 a	2.6 ± 0.08 a	60 ± 2.3 b

**Table:** Grain traits

	Protein content (%)	Test weight (g/L)	Diameter (mm)	Thousand kernel weight (g)	Hardness (HI)
2021					
Setodure	11.3 ± 0.12 a	863 ± 2.2 a	3.27 ± 0.02 a	52.5 ± 0.84 a	93 ± 1.2 a
*Psy-A1l* NIL	11.0 ± 0.14 a	864 ± 2.2 a	3.23 ± 0.02 a	51.6 ± 0.51 a	94 ± 0.8 a
*Psy-A1o* NIL	11.3 ± 0.18 a	862 ± 2.1 a	3.25 ± 0.02 a	52.1 ± 1.15 a	93 ± 0.6 a
*Psy-E1* NIL	12.1 ± 0.22 b	853 ± 2.3 b	3.19 ± 0.01 a	45.7 ± 0.56 b	93 ± 1.0 a
2022					
Setodure	12.2 ± 0.21 a	846 ± 3.4 a	3.24 ± 0.02 a	51.9 ± 0.68 a	91 ± 1.3 a
*Psy-A1l* NIL	11.9 ± 0.26 a	852 ± 2.9 a	3.26 ± 0.00 a	51.7 ± 0.22 a	90 ± 0.4 a
*Psy-A1o* NIL	11.8 ± 0.24 a	849 ± 2.5 a	3.22 ± 0.02 a	51.2 ± 0.78 a	90 ± 1.0 a
*Psy-E1* NIL	13.2 ± 0.25 b	831 ± 2.8 b	3.19 ± 0.02 a	45.3 ± 0.91 b	91 ± 0.6 a

*^a^* Sowing day was Nov. 11^th^ in each years. Heading and maturity date were calculated as the accumulated days after sowing. *^b^* Spikelet density = Spikelet number/Spike length Values are mean ± SE at 4 replicates (averages of 2 areas for spike number, 10 culms for culm length, and 8 spikes for spike traits). Within the same column, data marked with the same letter are not significant difference by Tukey’s HSD at *P* < 0.05.
