# Supplementary material for: Psy-E1 derived from Thinopyrum ponticum contributes strong yellowness to durum wheat but may cause yield loss in Japan
Source: Breed Sci. 2025 Mar 26;75(2):93–101. doi: 10.1270/jsbbs.24070 (PMC12395199; doi:10.1270/jsbbs.24070)
Supplement: Supplementary file 1 — Supplemental Figures [file 75_093_s1.pdf]

```
                                 20                    40                    60                    80
Psy-A1k  ATGGCCACCA CCGTCACGCT GCTGCTCGGG GCAGCCTCGT CCCCCGGCCC CGCCGCCGGT GATGGCGCCG CGCGGGACGG
Psy-A1l  ATGGCCACCA CCGTCACGCT GCTGCTCGGG GCAGCCTCGT CCCCCGGCCC CGCCGCCGGT GATGGCGCCG CGCGGGACGG
Psy-A1o  ATGGCCACCA CCGTCACGCT GCTGCTCGGG GCAGCCTCGT CCCCCGGCCC CGCCGCCGGT GATGGCGCCG CGCGGGACGG
Psy-E1   ATGGCCACCA CAGTCACGCT GCTGCTCGGG GCAGTCTCGT CCCCCGGCCC CGGCGCCGGT CTCGCCGCCG GAGATGCCGG
         ********** * ******** ********** **** ***** ********** ** *******    * *****      * ***
                      Psy-A1_GSP-F1 100                   120                   140                   160
Psy-A1k  CTTCCA-GTG CTCCCGC-CT GCTACC-CAA GAAGAAACAG CAGAGGCCGC GCTGGGTGCT CTGCTCGCTC AAGTACGGCT
Psy-A1l  CTTCCA-GTG CTCCCGC-CT GCTACC-CAA GAAGAAACAG CAGAGGCCGC GCTGGGTGCT CTGCTCGCTC AAGTACGGCT
Psy-A1o  CTTCCA-GTG CTCCCGC-CT GCTACC-CAA GAAGAAACAG CAGAGGCCGC GCTGGGTGCT CTGCTCGCTC AAGTACGGCT
Psy-E1   CCACCACGTG AGCCTGCACT GCTCCCGCCT GCGCGCGAGG AAGAGGCAGC CGTGGGTGCT CTGCTCGCTC AAGTACGGCT
         *  *** ***   ** ** ** *** ** *   *        *  ****** **   ******** ********** **********
                                180                   200                   220                   240
Psy-A1k  GCCTCGGCGT CGGCGAGCCG GGGGAGGCGG GCGGCCGGAG CGCGGCGTCG CCGGTGTACT CCAGCCTCAC CGTCAGCCCC
Psy-A1l  GCCTCGGCGT CGGCGAGCCG GGGGAGGCGG GCGGCCGGAG CGCGGCGTCG CCGGTGTACT CCAGCCTCAC CGTCAGCCCC
Psy-A1o  GCCTCGGCGT CGGCGAGCCG GGGGAGGCGG GCGGCCGGAG CGCGGCGTCG CCGGTGTACT CCAGCCTCAC CGTCAGCCCC
Psy-E1   GCCTCGGGGT CGGCGAGCCG GGGGAGGCGG CCGGCCGGAG CGCGGCGTCG CCGGTGTACT CCAGCCTCAC CGTCAGCCCC
         ******* ** ********** **********  ********* ********** ********** ********** **********
                                260                   280                   300                   320
Psy-A1k  GGAGGCGACG CCGCCGTCGC CGTCGTCTCG TCGGAGCAGA AGGTGTACGA CGTGGTGGTG AAGCAGGCGG CATTGCTCAA
Psy-A1l  GGAGGCGACG CCGCCGTCGC CGTCGTCTCG TCGGAGCAGA AGGTGTACGA CGTGGTGGTG AAGCAGGCGG CATTGCTCAA
Psy-A1o  GGAGGCGACG CCGCCGTCGC CGTCGTCTCG TCGGAGCAGA AGGTGTACGA CGTGGTCGTG AAGCAGGCGG CATTGCTCAA
Psy-E1   GGCGGCGACG CCGCCGTCGC CGTCGTCTCG TCCGAGCAGA AGGTGTACGA CGTGGTGGTG AAGCAGGCCG CATTGCTCAA
         ** ******* ********** ********** ** ******* ********** ****** *** ******** * **********
                                340                   360                   380                   400
Psy-A1k  GCGCCAGCTG CGCCCCTCGC AGCAACAG-- -CAGCAGGCG CCGCCAGCCG TCGCCAGGGA GCTGGACGCG CCGCGCGGCG
Psy-A1l  GCGCCAGCTG CGCCCCTCGC AGCAACAG-- -CAGCAGGCG CCGCCCGCCG TCGCCAGGGA GCTGGACGCG CCGCGCGGCG
Psy-A1o  GCGCCAGCTG CGCCCCCAGC AGCAGCAG-- GCGGCACA-G CCGCCCGCCG TCGCCAGGGA GCTGGACGCG CCGCGCGGCG
Psy-E1   GCGCCAGCTG CGCCCCTCGC AGCAGCAGCA GCAGCAGGCG CCGCCCGCCG TCGCCAGGGA GCTGGACGCG CCTCGCGGCG
         ********** ******  ** **** ***    * ***   * ***** **** ********** ********** ** *******
                                420                   440                   460                   480
Psy-A1k  GGCTCGGGGA GGCCTACGCC CGCTGCGGCG AGATCTGCGA GGAGTACGCC AAGACCTTCT ACCTCGGTAC GCCACTCCTT
Psy-A1l  GGCTCGGGGA GGCCTACGCC CGCTGCGGCG AGATCTGCGA GGAGTACGCC AAGACCTTCT ACCTCGGTAC GCCACTCCTT
Psy-A1o  GGCTCGGGGA GGCCTACGCC CGCTGCGGCG AGATCTGCGA GGAGTACGCC AAGACCTTCT ACCTCGGTAC GGTCCTCCTT
Psy-E1   GGCTCGGGGA GGCCTACGCC CGCTGCGGCG AGATCTGCGA GGAGTACGCC AAGACCTTCT ACCTCGGTAC GCCACCCCTT
         ********** ********** ********** ********** ********** ********** ********** *   * ****
                                500                   520                   540                   560
Psy-A1k  ----CGTGGA TACTCTGTTT TTCTTGAGCC ATGGTGGCAG GCTGCGTGCC AAGCC----- --------GG TGTTCCGGTG
Psy-A1l  ----CGTGGA TACTCTGTTT TTCTTGAGCC ATGGTGGCAG GCTGCGTGCC AAGCC----- --------GG TGTTCCGGTG
Psy-A1o  T---CATGCA TACTCTGTTT TTCTTGAGCC ATGGTGGCAA GCCG-ATG-- ---------- ---------- --TTCCGGTG
Psy-E1   CATGCATACT TACTCTGTTT TTCTTGAGCC TTGGTGGCAA GCTGCATGCC ATGCCAAGCC AGTGTTCTGA ACTTCTGATG
             * *    ********** **********  ********  ** *  **                          *** * **
                                                                              Psy-E1_GSP-F1
                                580                   600                   620                   640
Psy-A1k  ATCATGG--- -----AGCTC ACTCGTTCAT GTCTGGTCGT GCATGGCAGG GACCTTGCTG ATGACCGAGG AGCGGCGGCG
Psy-A1l  ATCATGG--- -----AGCTC ACTCGTTCAT GTCTGGTCGT GCATGGCAGG GACCTTGCTG ATGACCGAGG AGCGGCGGCG
Psy-A1o  ATCATGG--- -----AGCTC CTTCATTCAT GTCTGGTCGT GCATGGCAGG GACCTTGCTG ATGACCGAGG AGCGGCGGCG
Psy-E1   GTCATGGTCT CATGGAGCTC ACCCATTCAT GTCTGGTCGT GCATGGCAGG GACCTTGCTG ATGACAGAGG AGCGGCGGCG
          ******         *****    * ***** ********** ********** ********** ***** **** **********
                                660                   680                   700                   720
Psy-A1k  CGCCATATGG GCCATCTACG GTA----ATC TGAAAATTCA CCATGCCTGG TTTGGACCCT CCATTGTTGC TCCCCTGTTG
Psy-A1l  CGCCATATGG GCCATCTACG GTA----ATC TGAAAATTCA CCATGCCTGG TTTGGACCCT CCATTGTTGC TCCCCTGTTG
Psy-A1o  CGCCATATGG GCCATCTACG GTAATCAATC TGAAACCTCA CCAAGCCTGG CTTGGACCCT CCATTGTTGC TCCCCTGTTG
Psy-E1   CGCCATATGG GCCATCTACG GTAATCAATC TGAAAACGCA CCAAGCCTGG CTTGGACCCT CAATTGTTGC TCCCCTATTG
         ********** ********** ***    *** *****   ** *** ******  ********* * ******** ****** ***
                                740                   760                   780                   800
Psy-A1k  TGGTA---TC AGTATGTGTC ACACAGTGTT AGTTAGTGTC AGTAATGTGA CTGAAAATTC AGCTAGTTTC ATTCTCACTT
Psy-A1l  TGGTA---TC AGTATGTGTC ACACAGTGTT AGTTAGTGTC AGTAATGTAA CTGAAAATTC AGCTAGTTTC ATTCTCACTT
Psy-A1o  TAGTA---TC AGTATGTGTC ACACAGTGTC AGTTAGTTTC AGTAATGTGA CTGAAAATTC AGCTAGTTTC ATTCTCACTT
Psy-E1   TAGTAGTATC AGTATTTGTC ACACAGTGTC AGTTAGTTTC AGTAATGTGA CTGAAAATGG AGCCAGTTTC ATTTT-ACTT
         * ***   ** ***** **** *********  ******* ** ******** * ********   *** ****** *** * ****
                                820                   840                   860                   880
Psy-A1k  CAGACCGT-- -CAGAAAGGG CATGCCCACA TTTTGCATCA GTTAAATTGC TACATATTGT ATTTAACAGC AACTTGCAAG
Psy-A1l  CAGACCGT-- -CAGAAAGGG CATGCCCACA TTTTGCATCA GTTAAATTGC TACATATTGT ATTTAACAGC AACTTGCAAG
Psy-A1o  CAGACCGT-- -CAGAAAGGG CATGCCCACA TTTTGCATCA GTTAAATTGC TACATATTGT ATTTAACAGC AACTTGCAAG
Psy-E1   CAAACCATCA TCAGAAATGG CATGCCCACA TTTGGCATGA GT-AAATTGC TGTACATACT CCCTC----C ATCTCGAATT
         ** *** *    ****** ** ********** *** **** * ** ******* *  * **  *    *     * * ** * *  
                                900                   920                   940                   960
Psy-A1k  AATCTTCAAC ACTCCCCAAG AAAATTGGCC ACTTTAAAGT TAATGGTGTG AACTAGTTCT GGATGCGAAT AATGGCAAAT
Psy-A1l  AATCTTCACC ACTCCCCAAG AAAATTGGCC ACTTTAAAGT TAATGGTGTG AACTAGTTCT GGATGCGAAT AATGGCAAAT
Psy-A1o  AATCTTCAAC ACTCCCCAGG AAAATTGGCC ACTTTTCAGT TAATGGTGTG AACTAGTTCT GGATGCGAAT AATGGCAAAT
Psy-E1   AGTTGTCA-- -CTCCCCAAG AAAATTGCCC ACTTTTCGGT TAATGGCGTA GACTAGTTCT GGATGCGAAT AATGGCAAAT
         * *  ***    ****** * ******* ** *****   ** ****** **   ********* ********** **********
                      Psy-A1_STS-F2    980                  1000                  1020                  1040
Psy-A1k  AGAAACATTG CTGAACTTGC ATGCTATGTG TTTACAGATA CTCCTATATA CGTAGTATAG TCAGTGAAGA ATAAAGGGCT
Psy-A1l  AGAAACATTG CTGAACTTGC ATGCTATGTG TTTACAGATA CTCCTATATA CGTAGTATAG TCAGTGAAGA ATAAAGGGCT
Psy-A1o  AGAAACATTG CTGAACTTGC ATGCTATGTG TTTACAGATA CTCCTATATA CGTAGTATAG TCAGTGAAGA ATAAAGGGTT
Psy-E1   AGAAACATTG TGGAACTTGC ATGCTATATG TTTATAGATA CTCCTATATA ----GTATAG TCAGTGAAGA ATAAAGGGTT
         **********   ******** ******* ** **** ***** **********     ****** ********** ******** *
                               1060                  1080                  1100                  1120
Psy-A1k  CGTATACCAC TTTTTTATAT GCCATTATGT GTGGAAGCAT CAAATTAGGC TTTTTGTTGG CTAAATGGCT TCAATAGGAT
Psy-A1l  CGTGTCACAC TTTTTTATAT GCCATTTTGT TTGGAAGCAT CAAATTAGGC TTTTTGTTGG CTAAATGGCT TCAATAGGAT
Psy-A1o  CGTATAACAC TTTTTTATAT GCCATTATGT GTGGAAGCAT CAAATTAGGC TTTTTGTTGG CTAAATGGCT CCAATAGGAT
Psy-E1   CGTATAACAC TTTTTTATAT GCCATTATGT GTGGAAGCAT CAAATTAGGC TTTTTGTTGG CTAAATGGCT TCAATAGGAT
         *** *   *** ********** ****** ***  ********* ********** ********** **********  *********
                                                                              Psy-R5
                               1140                  1160                  1180                  1200
Psy-A1k  CAAAGTACAC GAGAAAAGGT TGCAAGAACA TATTCCTCAA ATTGCCTGGG GACATGAATC TGAGGGTACC GTCAGTTCTA
Psy-A1l  CAAAGTACAC GAGAAAAGGT TGCAAGAACA TATTCCTCAA ATTGCCTAAG GACATGAATC TGAGGGTACC GTCAGTTCTA
Psy-A1o  CAAAGTACAC GAGAAAAGGT TGCAAGAACA TATTCCTCAA ATTGCCTAAG GACATGAATC TGAGAGTACT GTCAGTTCTA
Psy-E1   CAAAGTACAT GAGAAAAGGT TGCAAGAACA TATTCCTCAA ATTGCCTAAG GACATGAATC TGAGGGTTCT GTCAGTTCTA
         *********  ********** ********** ********** *******  * ********** **** ** *  **********
```

```
                                            1220                           1240                           1260                           1280
                                               |                              |                              |                              |
Psy-A1k AATGAGATAT ACTCTAGGCA TCAATCACTT TCAGAATCTG ATGTATAGCA TCATT-GTTC AGTATGGTGC AGGAGGACAG
Psy-A1l AATGAGATAT ACTCTAGGCA TCAATCACTT TCAGAATCTG ATGTATAGCA TCATT-GTTC AGTATGGTGC AGGAGGACAG
Psy-A1o AATGAGATAT ACTCTAGGCA TCAATCACTT TCAGAATCTG ATGTATAGCA TCATT-GTTC AGTATGGTGC AGGAGGACAG
Psy-E1  AATGAGATAT ACTCTAGGCA TTGATCACTT TCAGAATCTG ATGTGTAGCA TCATTTGTGC AGTGTGGTGT AGGAGGACAG
        ********** ********** *  ******* ********** **** ***** ***** ** * *** *****  **********
                          1300       1320       1340       1360
Psy-A1k ACGAGCTGGT GGACGGTCCC AACGCGTCGC ACATCACGCC GCAGGCGCTG GACCGGTGGG AGAGGAGGCT GGAGGACCTC
Psy-A1l ACGAGCTGGT GGACGGTCCC AACGCGTCGC ACATCACGCC GCAGGCGCTG GACCGGTGGG AGAGGAGGCT GGAGGACCTC
Psy-A1o ACGAGCTGGT GGACGGTCCC AACGCGTCGC ACATCACGCC GCAGGCGCTG GACCGGTGGG AGAGGAGGCT GGAGGACCTC
Psy-E1  ACGAGCTGGT GGACGGGCCG AACGCGTCGC ACATCACGCC GCAGGCGCTG GACCGGTGGG AGAGGAGGCT GGAGGACCTC
        ********** ****** **  ********** ********** ********** ********** ********** **********
                          1380       1400       1420       1440
Psy-A1k TTCGCCGGGC GCCCCTACGA CATGCTCGAC GCCGCGCTCT CTGACACCAT CACCAAGTTC CCCATAGATA TTCAGGTATC
Psy-A1l TTCGCCGGGC GCCCCTACGA CATGCTCGAC GCCGCGCTCT CTGACACCAT CACCAAGTTC CCCATAGATA TTCAGGTATC
Psy-A1o TTCGCCGGGC GCCCCTACGA CATGCTCGAC GCCGCGCTCT CCGACACCAT CACCAAGTTC CCCATAGATA TTCAGGTACC
Psy-E1  TTCGCGGGGC GCCCCTACGA CATGCTCGAC GCCGCGCTCT CCGACACCAT CACCAAGTTC CCCATTGATA TTCAGGTACC
        ***** **** ********** ********** ********** * ******** ********** ***** **** ******** *
                          1460       1480       1500       1520
Psy-A1k AGCTTAGCCG GTGCATAATT GTTCAGTCCA CATTGTATGA TTCTGGTAGA ACAGAGTGGT GGTG---GAT ATTCCCTGTC
Psy-A1l AGCTTAGCCG GTGCATAATT GTTCAGTCCA CATTGTATGA TTCTGGTAGA ACAGAGCGGT GGTG---GAT ATTCCCTGTC
Psy-A1o AGCTC-GCCG GTGCATAATT GTTCAGTCCA CATTGTATGA TTCCGGTAGA ACAGAGTGGT AGTG---GAT ATTCCCTGTC
Psy-E1  AGCTC-GCCG GTGCATAATT GTTCAGTCCA CATTGTATGA TTCTGGTAGA ACAGAGTGGT GGTGGTGGAT ATTCCCTGTC
        ****  **** ********** ********** ********** *** ****** ****** ***  ***   *** **********
                          1540       1560       1580       1600
Psy-A1k AGCATCAAAT TGCCCCTAGA CCTCACAATC TCAGTGCAAG ATGAC----- ---------- -------CGG AAAGTCGATG
Psy-A1l AGCATCAGAT TGCCCCTAGA CCTCACAATC TAAGTGCAAG ATGAC----- ---------- -------CAG AAAGTCGATG
Psy-A1o AGCATCAGAT T-CCCCCAGA CCTCACAATC TCAGTGCAAG ATGACTAGCT AGGCCTTCTA AGTTGACCAG AAAGTCGATG
Psy-E1  AGCATCAGAT T-CCCCCAGA CCTCACAATC TCAGTGCAAG ATGACTAGCT AGGCCTTCTA AGTTGACCAA TAAGTCGATG
        ******* ** * **** *** ********** * ******** *****                       *    *********
                                                                             Psy-E1_GSP-R
                          1620       1640       1660       1680
Psy-A1k ATTG-GTCAA AATTGTTTCG TTTGTCGGCC TTTGGTTAGT CTCTGATGCT GTTGTTGAGC CGTATGAACT TTTCACACAT
Psy-A1l ATTG-GTCAA AATTGTTTCG TTTGTCGGCC TTTTGTTAGT CTCTGATGCT GTTGTTGAGC CGTATGAACT TTTCACACAT
Psy-A1o ATTTTGTCAA AATTATTTCG TTTGTCGGCC TTTTATTAGT CTCTGATGCT GTAATTGAGC TGTATGAACT TTTCACACAT
Psy-E1  GTTG-GTCAA AATTGTTTCG TTTGTCGGCC TTTTATTAGT CCCTGATGCT GTTATTGAGA TGTATGAATT TTTCACACAT
         **  ***** **** ***** ********** ***  ***** * ******** **  *****   ******* * **********
                          1700       1720       1740       1760
Psy-A1k TGTAGTGGGG GCTTATCCTG TTGACTAGAC GCTATAGTGG GAATCGTCTG GTCAAAGATA TGTTTAATCA AAGTGGGGAA
Psy-A1l TGTGGTGGGG GCTTATCCAG TTGATTAGAC GCTATAGTGG GAATCGTCTG GTCAAAGATA TGTTTAATCA AAGTGGGGAA
Psy-A1o TGTAGTGGGG GCTTATCCAG TTGACTAGAC G-TATAGTGG GAATCATCTG GTAAAAGATA TGTTTAATCA ATGTGGGGAA
Psy-E1  TGTAGTGGGG GCTTATCCAG TTGACTAGGC GCTGTAGTGG GAATCATCTG GTCAAAGATA TGTTTAATCA AAGTGGGGAA
        *** ****** ******** * **** *** * * * ****** ***** **** ** ******* ********** * ********
                          1780       1800       1820       1840
Psy-A1k ATTATAGGGA GAACTTTTCA GTAAGTATTA TTCCCGAGCA TACCTAGAAC AAACAGGAAC TTGGAAATGA TGTAGAAGGA
Psy-A1l ATTATAGGGA GAACTTTTCA GCAAGTATTA TTCCTGAACA TACCTAGAAC AAACAGCAAC TTGGAAATGA TGTAGAAGGA
Psy-A1o ATTATAGGCA GAACTTTTCA GTAAGAATTA G--------- ---------- ---------- ---------- ----------
Psy-E1  ATTATAGGGA GAACTTTTCA ATAAAAATTA GTCCTGAACA TAGTAAGAAC AAACAGGAAC TTGGAAATGC TGTAGAAGGA
        ******** * **********   **  ****
                          1860       1880       1900       1920
Psy-A1k GAAATGAAAT CAGGGAGAGT CCAAGTAACT CCAGACCTGA ATTATACATC ATGAAAATGT ACCTGGTCAC TC--TTTTTT
Psy-A1l GAAATGAAAT CAGGGAGAGT CCAAGTAACT CCAGACCTGA ATTATACATC ATGAAAATGT ACTTGGTCAC TC--TTTTTT
Psy-A1o ---------- ---------- ---------- ---------- ---------- ---------- ---------- ----------
Psy-E1  GAACTGAAAT CAGGAAGAGT CGAAGTAATT CCAGACCAGA ATACTACATC ATGAAAATGT TCTTGGTCAC TCTGTTTTTT
                          1940       1960       1980       2000
Psy-A1k ACTGGGCTTT GTGGTTTTCG GCCTAATTTC CCATATAAAC CGCGTTACTC TCTATTTCTT ---------- ----------
Psy-A1l ACTGGGCTTT GTGGTTTTCG GCCTAATTTC CCATATAAAC CGCGTTACTC TCTATTTCTT TCCTTTTGTT T-CGATGAAA
Psy-A1o ---------- ---------- ---------- ---------- ---------- ---------- TTCTTTTTCT CATAGTGAAA
Psy-E1  ACTGGGCTTT GTGGTTTTTG GCCTAATTTT TCGTATAAAC CGCGTTACTC TGTATTTCTT TTCTTTTTCT CATAGTGAAA
                          2020       2040       2060       2080
Psy-A1k ---GCAGAGC TCCTGACTTG CATTGTAAAA AAAAA-GGTC TTGGTCGTTC TTAGCACTAC TACTTATGAA AAATATTATT
Psy-A1l TCAGCAGAGC TTCTGCCTTG CATTGTAAAA AAAAAAGGTC TTGGTCGTTC TTAGCACTAC TACTTATGAA AAATATTATT
Psy-A1o TCGGCAGTGA TCCTGCCTTG CATTGTAAAA AAAA--GGTC TTGCAGAGTA CTACTTATGA -ACT------ --ATATTATT
Psy-E1  TCGGCAGTGA TCCTGCCTTG CATTGTAAAA AAAAA-GGTC TTGGTCCTTG TTAGCAATAC TACTTG---- -GGAACTAGT
           **** *  * *** **** ********** ****  **** ***     *   **    *    ***           * ** *
                          2100       2120       2140       2160
Psy-A1k GATTTTCT-A AATGACCAAT TACTTTTACA TATGCCAGCC CTTCAAGGAC ATGATCGACG GGATGCGGAC GGACCTCAAG
Psy-A1l GATTTTCT-A AATGACCAAT TACTTTTACA TATGCCAGCC CTTCAAGGAC ATGATCGACG GGATGCGGAC GGACCTCAAG
Psy-A1o GATATTCTAA AATGACCAAT TATTTTTACA TATGCCAGCC CTTCAAGGAC ATGATCGACG GGATGCGGAC AGACCTCAAG
Psy-E1  AATAGTTTTA AATGACTAAT TATTCGTACA TATGCCAGCC CTTCAAGGAC ATGATCGACG GGATGCGGAC GGACCTCAAG
         **  * * * ****** *** ** *  **** ********** ********** ********** **********  *********
                          2180       2200       2220       2240
Psy-A1k AAGGCGAGGT ACAAGAACTT TGACGAGCTC TACATGTACT GCTACTATGT TGCCGGCACC GTGGGGTTGA TGAGCGTCCC
Psy-A1l AAGGCGAGGT ACAAGAACTT TGATGAGCTC TACATGTACT GCTACTATGT TGCCGGCACC GTGGGGTTGA TGAGCGTCCC
Psy-A1o AAAGCAAGAT ACAAGAACTT CGACGAGCTC TACATGTACT GCTACTATGT CGCGGGTACG GTGGGGCTGA TGAGCGTCCC
Psy-E1  AAGGCGAGGT ACAAGAACTT TGACGAGCTC TACATGTACT GCTACTACGT TGCGGGCACC GTTGGGCTGA TGAGTGTCCC
        ** ** ** * **********  ** ****** ********** ******* **  ** ** **  ** *** *** **** *****
                          2260       2280       2300       2320
Psy-A1k GGTGATGGGC ATTGCGCCCG ACTCCAAGGC GACAGCTGAG AGCGTCTATG GCGCCGCTCT GGCTCTCGGG CTCGCGAACC
Psy-A1l GGTGATGGGC ATTGCACCCG ACTCCAAGGC GACAGCTGAG ACCGTCTATG GCGCCGCTCT GGCTCTCGGG CTCGCGAACC
Psy-A1o GGTGATGGGC ATTGCGCCCG AGTCCAAGGC GACGGCGGAG AGCGTCTACG GCGCCGCTCT GGCTCTCGGG CTGGCGAACC
Psy-E1  AGTGATGGGC ATTGCGCCCG AGTCCAAGGC GACGGCCGAG AGCGTCTACG GCACCGCTCT GGCTCTCGGG CTGGCGAACC
         ********* ***** **** * ******** *** ** *** * ****** * ** ******* ********** ** *******
                          2340       2360       2380       2400
Psy-A1k AGCTCACCAA CATACTCAGG GATGTCGGAG AAGAGTAAGC CACTCACTCA CTACCAATAC AATGCAATAG TTTTCCCTTG
Psy-A1l AGCTCACCAA CATACTCAGG GATGTCGGAG AAGAGTAAGC CACTCACTCA CTACCAATAC AATGCAATGG TTTTCCCTTG
Psy-A1o AGCTCACCAA CATACTCAGG GATGTTGGAG AAGAGTAAGC CACCCACTGA TTACAACTAA AATGCAACGG TTTTCCCTTC
Psy-E1  AGCTCACCAA CATACTCAGG GATGTTGGAG AAGAGTAAGC AACTCACTCA CTATAC---A GATGCAATGG TTTTCTCTT-
        ********** ********** ***** **** **********  ** **** *  **         ******  * ***** ***
```

```
                          2420                  2440                  2460                  2480
Psy-A1k  TAAAATCATT TTTTTTAGAA AAGGAGCATG ACCCCCGGCC TCTGCATCTG AGAGATGCAT ACGGCCACTT TATTGATTAT
Psy-A1l  TAAAATCAT- ---------- ---------- ---------- ---------- ---------- ---------- ----------
Psy-A1o  CTAA------ ---------- ---------- ---------- ---------- ---------- ---------- ----------
Psy-E1   ---------- ---------- ---------- ---------- ---------- ---------- ---------- ----------

                          2500                  2520                  2540                  2560
Psy-A1k  TCTCAGGACC TTACAAAGTA TTACAACAAT GAGCCTGAAT CCACCATCTT GACAACACAT GCCGCTACTC CTATCCAAAA
Psy-A1l  ---------- ---------- ---------- ---------- ---------- ---------- ---------- ----------
Psy-A1o  ---------- ---------- ---------- ---------- ---------- ---------- ---------- ----------
Psy-E1   ---------- ---------- ---------- ---------- ---------- ---------- ---------- ----------

                          2580                  2600                  2620                  2640
Psy-A1k  TGATGAAGGG GTGCTAGCTG GGCCACTACC CAAACCACTC ACCAAAGCCT AACATCAAAA GCCGGAAACC GAAACATATT
Psy-A1l  ---------- ---------- ---------- ---------- ---------- ---------- ---------- ----------
Psy-A1o  ---------- ---------- ---------- ---------- ---------- ---------- ---------- ----------
Psy-E1   ---------- ---------- ---------- ---------- ---------- ---------- ---------- ----------

                          2660                  2680                  2700                  2720
Psy-A1k  CGGAAGCCCC AGCCGAGCCA CATACCGGGT CTGGGCACAA TCCGGTCAGA CGCACTCGTG TGTCGTCGCC GCCATCTTCC
Psy-A1l  ---------- ---------- ---------- ---------- ---------- ---------- ---------- ----------
Psy-A1o  ---------- ---------- ---------- ---------- ---------- ---------- ---------- ----------
Psy-E1   ---------- ---------- ---------- ---------- ---------- ---------- ---------- ----------

                          2740                  2760                  2780                  2800
Psy-A1k  ACAGGTCCGT CTTCAGATCA TATTGAGGCT TCTACCTTGT CTGGCCACTC TACCATCGAC GTCACCATGA CGCCAGACAG
Psy-A1l  ---------- ---------- ---------- ---------- ---------- ---------- ---------- ----------
Psy-A1o  ---------- ---------- ---------- ---------- ---------- ---------- ---------- ----------
Psy-E1   ---------- ---------- ---------- ---------- ---------- ---------- ---------- ----------

                          2820                  2840                  2860                  2880
Psy-A1k  CAACCTCCTC CTGCGCGAGT CCATCTCCGT GCATCGGGCG GCGAGCCTCC GCAGCGCCAT GCCGCCGATC TTCGCCGCCA
Psy-A1l  ---------- ---------- ---------- ---------- ---------- ---------- ---------- ----------
Psy-A1o  ---------- ---------- ---------- ---------- ---------- ---------- ---------- ----------
Psy-E1   ---------- ---------- ---------- ---------- ---------- ---------- ---------- ----------

                          2900                  2920                  2940                  2960
Psy-A1k  TCAATGAGTG AGATGAAGTA CCGCTCCACC ACGGCATGTA CAAGGTGACG AAGGGCGAGG TCCCCATCGG AGACACGGGC
Psy-A1l  ---------- ---------- ---------- ---------- ---------- ---------- ---------- ----------
Psy-A1o  ---------- ---------- ---------- ---------- ---------- ---------- ---------- ----------
Psy-E1   ---------- ---------- ---------- ---------- ---------- ---------- ---------- ----------

                          2980                  3000                  3020                  3040
Psy-A1k  GGAAGAGAAG CACCGCAGCC CCGAGACACT GCCCGGAGTT GCGACGCAGT AGATCAGGCG GGCCGTCACC AGGAACCAGA
Psy-A1l  ---------- ---------- ---------- ---------- ---------- ---------- ---------- ----------
Psy-A1o  ---------- ---------- ---------- ---------- ---------- ---------- ---------- --GAAC----
Psy-E1   ---------- ---------- ---------- ---------- ---------- ---------- ---------- --GTAC----

                          3060                  3080                  3100                  3120
Psy-A1k  CAAGCACGCC ATGCACCCCA GCATCCCCAT CCCCATGCCC ATCCCTTGTA AAATCATGAT ATGGACATTT CGAGATAGCA
Psy-A1l  ---------- ---------- ---------- ---------- ---------- -------GAT ATGGACATTT TGAGATAGCA
Psy-A1o  ---------- ---------- ---------- ---------- ---------- -AGTCATGAT ATGAGTATT- -AAAATAGCA
Psy-E1   ---------- ---------- ---------- ---------- ---------- -AATCATGAT ATGGACATTT TCAGATAGCA
                                                                      *** ***   ***    * ******
                          3140                  3160                  3180                  3200
Psy-A1k  CTATCCTGAA TTTTTTGGTG TTTCGAATAA TTTCCC---A GTGCAAGAAG AGGAAGGATA TATCTACCAC AAGACGAGCT
Psy-A1l  CTATCCTGAA TTTTCTGGTG TTTCGAATAA TTTCCC---A GTGCAAGAAG AGGAAGGATA TATTTGCCGC AAGACGAGCT
Psy-A1o  CTGTT-TGAA ATTTTGTGTG TTTGCAATAT TTCTTTTCCA GTGCGCGAAG AGGAAGGATA TATTTGCCGC AAGACGAGCT
Psy-E1   CTATCCTGAA TTTTTGGGTG CTTCGATTAA TTTCTT---A GTGCGAGAAG AGGGAGGATA TATTTGCCGC AAGACGAGCT
         ** *  ****  ***   ***  **  * **  **       * ****  **** *** ****** *** * ** * **********
                          3220                  3240                  3260                  3280
Psy-A1k  CGCGGAGGCG GGGCTCTCCG ATGAAGACAT CTTCAAAGGT GTCGTCACCG ACAAGTGGAG AAAATTCATG AAGAGGCAGA
Psy-A1l  CGCGGAGGCA GGGCTCTCCG ACGAAGACAT CTTCAAAGGA GTCGTCACCG ACAAGTGGAG AAAATTCATG AAGAGGCAGA
Psy-A1o  TGCGGAGGCA GGGCTCTCCG ATGAAGACAT CTTCAAAGGA GTCGTCACCG ACAAGTGGAG GAAATTCATG AAGAGGCAGA
Psy-E1   TGCGGAGGCA GGGCTCTCCG ATGAAGACAT CTTCAAAGGA GTAGTCACCG ACAAGTGGAG GAAATTCATG AAGAGGCAGA
          ********  ********** * ******** *********  ** ******* **********  ********* **********
                          3300                  3320                  3340                  3360
                    <---------------- Psy-A1_STS-R
Psy-A1k  TCAAGAGGGC GAGGATGTTC TTCGAGGAGG CGGAGCGAGG GGTGACTGAG CTTAGGAAGG AGAGCCGGTG GCCGGTAAGT
Psy-A1l  TCAAGAGGGC GAGGATGTTC TTCGAGGAGG CGGAGCGAGG GGTGACTGAG CTTAGGAAGG AGAGCCGGTG GCCGGTAAGT
Psy-A1o  TCAAGAGGGC GAGGATGTTC TTCGAGGAGG CGGAGCGAGG GGTGACCGAG CTTAGGAAGG AGAGCCGGTG GCCGGTAAGT
Psy-E1   TCAAGAGGGC GAGGATGTTC TTTGAGGAGG CGGAGCGAGG GGTGACCGAG CTCAGGAAGG AGAGCCGGTG GCCGGTAAGT
         ********** ********** ** ******* ********** ****** *** ** ******* ********** **********
                          3380                  3400                  3420                  3440
Psy-A1k  GCCC---AGC CACGACTTGA ATGTGAAACA AAACTACATA TTGATCTCAC ATCATTGTTA ATTATCAGTA GCAAAAATGA
Psy-A1l  GCCCTAAAGC CACGCCTTGA ATGTGAAACA AAACTACATA TTGATCTCTC ATCATTGTTA ATTATCCGTA GCAAAAATGA
Psy-A1o  GCCCTAATAC CACAACTTGA TAACCAAAAA GAAGTACATA TAGAGTTCTC ATCGATGTTA ATTATTAGTA ACAACGAAGG
Psy-E1   GCCTTA-CGC CACGACTTGA ATGGCAAACA GAATTGCATA TAGATCTCTC ATCAGTGTTA ATTATCCGTG GCAAAAACAA
         ***      * ***  *****      *** *  ** * **** * **  ** * ***  ***** *****  **   ***  *
                          3460                  3480                  3500                  3520
Psy-A1k  TGCTACGTGT AGTTCGTG-- ---------- ---------- --GGGGGTCG CCTCCCCCCA CCCTTGGTAT AATAATCATT
Psy-A1l  TGCTACGTGT AGTTCGTG-- ---------- ---------- --GGGGGTCG CCTCCCCCCA CCCTTGGTAT AATAATCATT
Psy-A1o  TGCCATGTGT AGTTCAAGAC CCGCAAAAAA AAATGTGTCA AGGGGGGTCG CCGCCCCTCA CC-TTGGTAT AATCATTGAA
Psy-E1   AGCGGT---T AGTTCATG-- ---------- ---------- --GGGGGGCA CCGCACC-CA CC-TTGGTAT AAGCATTGAA
          **      * *****  *                          ***** *  ** * ** ** ** ******* **  **
                          3540                  3560                  3580                  3600
Psy-A1k  GAAAAAA-AT TAGGGGCTCA AATGGAAGA- ---------- ------TAAT ATGGTTTTGC ATTGCATTGC AATTGCAGGT
Psy-A1l  GAAAAAA-AT TAGGGGCTCA AATGGAAGA- ---------- ------TAAT ATGGTTTTGC ATTGCATTGC AATTGCAGGT
Psy-A1o  GGAAAAATAT TAGGGGCTCA AATAGAAGAA AGTATCACTT GAGTGATAAT ATGGTTTTGC ATTG-----C AATTGCAGGT
Psy-E1   AAAAAA---- TAGGGTCTCA AATAGAAGAA -GTCTCAGTA GAGTGATAAT ATGGTTTTGC ATTGCATTGC AATTGCAGGT
           ****     ***** **** *** *****                  **** ********** ****     * **********
```

```
                                        3620                                    3640                                    3660                                    3680
                                           |                                       |                                       |                                       |
Psy-A1k  TTGGGCCTCT CTGTTGTTGT ACCGGCAGAT CCTTGACGAG ATCGAAGCGA ATGACTACAA CAACTTCACC AAGAGGGCCT
Psy-A1l  TTGGGCCTCT CTGTTGTTGT ACCGGCAGAT CCTCGACGAG ATCGAAGCGA ATGACTACAA CAACTTCACC AAGAGGGCCT
Psy-A1o  TTGGGCCTCT TTGTTGCTAT ACCGGCAGAT CCTCGATGAG ATCGAAGCGA ACGACTACAA CAACTTCACC AAGAGGGCCT
Psy-E1   TTGGGCCTCT CTGTTGTTGT ACCGGCAGAT CCTCGATGAG ATCGAGGCGA ACGACTACAA CAACTTCACC AAGAGGGCCT
         ********** ***** * * ********** *** ** *** ***** **** * ******** ********** **********
                                        3700                                    3720                                    3740                                    3760
                                           |                                       |                                       |                                       |
Psy-A1k  ATGTTGGGAA GGCGAAAAAG GTGCTTGCGC TCCCTGTCGC GTACGGGAGA TCGCTGCTCT TACCGTATTC ACTGAGAAAT
Psy-A1l  ATGTTGGGAA GGCGAAAAAG GTGCTTGCGC TGCCTGTCGC GTACGGGAGA TCGCTGCTCT TACCGTATTC ACTGAGAAAT
Psy-A1o  ATGTTGGGAA GGCGAAGAAG GTGCTGGCGC TCCCTGTCGC GTACGGGAGA TCGCTGCTCT TACCGTATTC ACTGAGAAAT
Psy-E1   ATGTTGGGAA GGCGAAGAAG GTGCTGGCGC TCCCCGTTGC GTATGGGAGA TCGCTGCTCT TACCGTATTC ACTGAGAAAT
         ********** ****** *** ***** **** * ** ** ** *** ****** ********** ********** **********
                     3772
                        |
Psy-A1k  AACCAGACCT AG  3651
Psy-A1l  AACCAGACCT AG  2967
Psy-A1o  AACCAGACCT AG  2804
Psy-E1   AACCAGACCT AG  3022
         ********** **
```

Supplemental Fig.1 Alignment of *Psy-A1* and *Psy-E1* sequences. The sequences of *Psy-A1k*, *Psy-A1l*, *Psy-A1o* and *Psy-E1* were corresponded to LC778135, EU096090, FJ234424 and EU096095, respectively. Start and stop codon were shown in yellow and primers positions were shown in red.

```
                             20                    40                    60
Psy-A1k   MATTVTLLLG AASSPGPAAG DGAARDGFQC SRLLPKKKQ- QRPRWVLCSL KYGCLGVGEP
Psy-A1l   MATTVTLLLG AASSPGPAAG DGAARDGFQC SRLLPKKKQ- QRPRWVLCSL KYGCLGVGEP
Psy-A1o   MATTVTLLLG AASSPGPAAG DGAARDGFQC SRLLPKKKQ- QRPRWVLCSL KYGCLGVGEP
Psy-E1    MATTVTLLLG AVSSPGPGAG LAAGDAGHHV SLHCSRLRAR KRQPWVLCSL KYGCLGVGEP
          ********** *.*****.**  .*.  *.:  *   .: :   :*  ****** **********
                             80                   100                   120
Psy-A1k   GEAGGRSAAS PVYSSLTVSP GGDAAVAVVS SEQKVYDVVV KQAALLKRQL RP-SQQQQQA
Psy-A1l   GEAGGRSAAS PVYSSLTVSP GGDAAVAVVS SEQKVYDVVV KQAALLKRQL RP-SQQQQQA
Psy-A1o   GEAGGRSAAS PVYSSLTVSP GGDAAVAVVS SEQKVYDVVV KQAALLKRQL RP-QQQQAAQ
Psy-E1    GEAAGRSAAS PVYSSLTVSP GGDAAVAVVS SEQKVYDVVV KQAALLKRQL RPSQQQQQQA
          ***.****** ********** ********** ********** ********** ** .***
                            140                   160                   180
Psy-A1k   PPAVARELDA PRGGLGEAYA RCGEICEEYA KTFYLGTLLM TEERRRAIWA IYVWCRRTDE
Psy-A1l   PPAVARELDA PRGGLGEAYA RCGEICEEYA KTFYLGTLLM TEERRRAIWA IYVWCRRTDE
Psy-A1o   PPAVARELDA PRGGLGEAYA RCGEICEEYA KTFYLGTLLM TEERRRAIWA IYVWCRRTDE
Psy-E1    PPAVARELDA PRGGLGEAYA RCGEICEEYA KTFYLGTLLM TEERRRAIWA IYVWCRRTDE
          ********** ********** ********** ********** ********** **********
                            200                   220                   240
Psy-A1k   LVDGPNASHI TPQALDRWER RLEDLFAGRP YDMLDAALSD TITKFPIDIQ PFKDMIDGMR
Psy-A1l   LVDGPNASHI TPQALDRWER RLEDLFAGRP YDMLDAALSD TITKFPIDIQ PFKDMIDGMR
Psy-A1o   LVDGPNASHI TPQALDRWER RLEDLFAGRP YDMLDAALSD TITKFPIDIQ PFKDMIDGMR
Psy-E1    LVDGPNASHI TPQALDRWER RLEDLFAGRP YDMLDAALSD TITKFPIDIQ PFKDMIDGMR
          ********** ********** ********** ********** ********** **********
                            260                   280                   300
Psy-A1k   TDLKKARYKN FDELYMYCYY VAGTVGLMSV PVMGIAPDSK ATAESVYGAA LALGLANQLT
Psy-A1l   TDLKKARYKN FDELYMYCYY VAGTVGLMSV PVMGIAPDSK ATAETVYGAA LALGLANQLT
Psy-A1o   TDLKKARYKN FDELYMYCYY VAGTVGLMSV PVMGIAPESK ATAESVYGAA LALGLANQLT
Psy-E1    TDLKKARYKN FDELYMYCYY VAGTVGLMSV PVMGIAPESK ATAESVYGTA LALGLANQLT
          ********** ********** ********** *******:** ****:***:* **********
                            320                   340                   360
Psy-A1k   NILRDVGEDA RRGRIYLPQD ELAEAGLSDE DIFKGVVTDK WRKFMKRQIK RARMFFEEAE
Psy-A1l   NILRDVGEDA RRGRIYLPQD ELAEAGLSDE DIFKGVVTDK WRKFMKRQIK RARMFFEEAE
Psy-A1o   NILRDVGEDA RRGRIYLPQD ELAEAGLSDE DIFKGVVTDK WRKFMKRQIK RARMFFEEAE
Psy-E1    NILRDVGEDA RRGRIYLPQD ELAEAGLSDE DIFKGVVTDK WRKFMKRQIK RARMFFEEAE
          ********** ********** ********** ********** ********** **********
                            380                   400                   420
Psy-A1k   RGVTELRKES RWPVWASLLL YRQILDEIEA NDYNNFTKRA YVGKAKKVLA LPVAYGRSLL
Psy-A1l   RGVTELRKES RWPVWASLLL YRQILDEIEA NDYNNFTKRA YVGKAKKVLA LPVAYGRSLL
Psy-A1o   RGVTELRKES RWPVWASLLL YRQILDEIEA NDYNNFTKRA YVGKAKKVLA LPVAYGRSLL
Psy-E1    RGVTELRKES RWPVWASLLL YRQILDEIEA NDYNNFTKRA YVGKAKKVLA LPVAYGRSLL
          ********** ********** ********** ********** ********** **********
                 430
Psy-A1k   LPYSLRNNQT 428
Psy-A1l   LPYSLRNNQT 428
Psy-A1o   LPYSLRNNQT 428
Psy-E1    LPYSLRNNQT 430
          **********
```

Supplemental Fig.2 Alignment of deduced amino acid sequences of Psy-A1 and Psy-E1. The amino acid sequences of Psy-A1l, Psy-A1o and Psy-E1 were corresponded to ABW80608, ACO07290 and ABW80613, respectively. Amino acid sequence of Psy-A1k in ‘Setodure’ was substituted ACQ59133 as identical amino acid sequence of Psy-A1k in *T.diccocoides.*

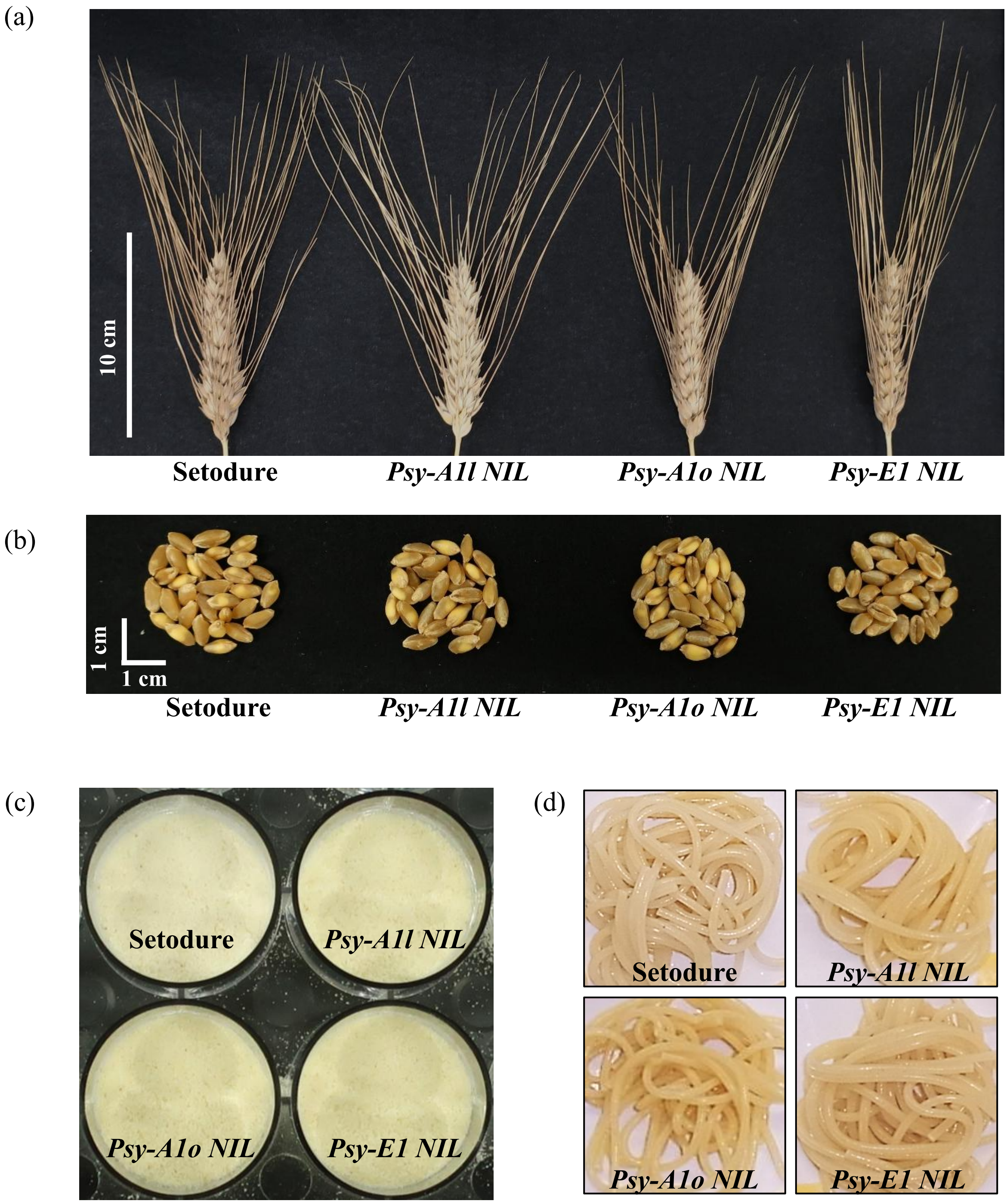

Supplemental Fig.3 Representative photographs among 'Setodure' and NILs. (a) spikes, (b) seeds, (c) semolina flour and (d) boiled spaghetti were shown. Scale bars were 10 cm in (a) and 1 cm in (b).
