# Supplementary material for: Psy-E1 derived from Thinopyrum ponticum contributes strong yellowness to durum wheat but may cause yield loss in Japan
Source: Breed Sci. 2025 Mar 26;75(2):93–101. doi: 10.1270/jsbbs.24070 (PMC12395199; doi:10.1270/jsbbs.24070)
Supplement: Supplementary file 2 — Supplemental Table [file 75_093_s2.pdf]

doi: 10.1270/jsbbs.24070

Supplemental Table 1 Sequences of primers used in this study.

PCR target	Primer name	Sequence 5′–3′	Reference
For DNA sequencing of *Psy-A1* in 'Setodure'			
Psy-A1 ORF	5′ UTR-F	TCACACGAGAGTGGTGAATCC	Designed in this study
	3′ UTR-R	ATCTATCCTAACCTGACCATCTTCATC	Designed in this study
DNA sequencing	Seq1	ATGGCCACCACCGTCACG	Designed in this study
	Seq2	TCCTCGAAGAACATCCTC	Singh et al. (2009)
	Seq3	CATGCTATGTGTTTACAGATACTCCTATATACG	Designed in this study
	Seq4	TCCCCAGGCAATTTGAGGAA	Designed in this study
	Seq5	CGGTGGGAGAGGAGGCTGGA	Designed in this study
	Seq6	CAGCCGTACTTGAGCGAGCA	Designed in this study
	Seq7	CATGACGCCAAACAGCAACC	Designed in this study
For DNA marker-assisted selection of *Psy1* genes and *Lr19* gene for NIL establishment and segregation test			
Psy-A1	Psy-A1_STS-F2	Same as above in Seq3	Designed in this study
	Psy-A1_STS-R	Same as above in Seq2	Singh et al. (2009)
Psy-E1	Psy-E1_GSP_F1	CAGTGTTCTGAACTTCTGATGGTCATGGTC	Designed in this study
	Psy-E1_GSP_R	TCGACTTATTGGTCAACTTAGAAGGCCTAGC	Designed in this study
Psy1 (*Psy-A1* and *Psy-E1*)	Psy-A1_GSP_F1	CGCCTGCTACCCAAGAAGAAACAGC	Designed in this study
	Psy-E1_GSP_F1	Same as above	Designed in this study
	Psy_R5	AGCCATTTAGCCAACAAAAAGCCTA	Designed in this study
Lr19	Lr19_AG15_F4	CAGCTACGTGCATCCCTTTCTT	Gennaro et al. (2009)
	Lr19_R2	CCAGCTGCTTCTTCACCGTCGTCCTT	Designed in this study
Waxy-B1	BDFL	CTGGCCTGCTACCTCAAGAGCAACT	Saito et al. (2009)
	BRC1	GGTTGCGGTTGGGGTCGATGAC	Saito et al. (2009)
	BFC	CGTAGTAAGGTGCAAAAAAGTGCCACG	Saito et al. (2009)
	BRC2	ACAGCCTTATTGTACCAAGACCCATGTGTG	Saito et al. (2009)

Supplemental literature cited

Saito, M., P. Vrinten, G. Ishikawa, R. Graybosch and T. Nakamura (2009) A novel codominant marker for selection of the null *Wx-B1* allele in wheat breeding programs. Mol Breed 23: 209–217.
